# Preeclamptic placentae release factors that damage neurons: implications for foetal programming of disease

**DOI:** 10.1042/NS20180139

**Published:** 2018-10-12

**Authors:** Hannah Scott, Tom J. Phillips, Greer C. Stuart, Mark F. Rogers, Bruno R. Steinkraus, Simon Grant, C. Patrick Case

**Affiliations:** 1 School of Clinical Sciences, University of Bristol, Learning & Research Building, Southmead Hospital, Bristol BS10 5NB, U.K.; 2 UK Dementia Research Institute, Cardiff University, Hadyn Ellis Building, Maindy Road, Cardiff CF24 4HQ, U.K.; 3 Department of Obstetrics, Southmead Hospital, Bristol BS10 5NB, U.K.; 4 Intelligent Systems Laboratory, University of Bristol, Merchant Venturers Building, Woodland Road, Bristol BS8 1UB, U.K.; 5 Weatherall Institute of Molecular Medicine, Radcliffe Department of Medicine, University of Oxford, John Radcliffe Hospital, Oxford OX3 9DS, U.K.

**Keywords:** astrocytes, glutamate, microRNA, neurodevelopmental disorders, neurons, preeclampsia

## Abstract

Prenatal development is a critical period for programming of neurological disease. Preeclampsia, a pregnancy complication involving oxidative stress in the placenta, has been associated with long-term health implications for the child, including an increased risk of developing schizophrenia and autism spectrum disorders in later life. To investigate if molecules released by the placenta may be important mediators in foetal programming of the brain, we analysed if placental tissue delivered from patients with preeclampsia secreted molecules that could affect cortical cells in culture. Application of culture medium conditioned by preeclamptic placentae to mixed cortical cultures caused changes in neurons and astrocytes that were related to key changes observed in brains of patients with schizophrenia and autism, including effects on dendrite lengths, astrocyte number as well as on levels of glutamate and γ-aminobutyric acid receptors. Treatment of the placental explants with an antioxidant prevented neuronal abnormalities. Furthermore, we identified that bidirectional communication between neurons and astrocytes, potentially via glutamate, is required to produce the effects of preeclamptic placenta medium on cortical cells. Analysis of possible signalling molecules in the placenta-conditioned medium showed that the secretion profile of extracellular microRNAs, small post-transcriptional regulators, was altered in preeclampsia and partially rescued by antioxidant treatment of the placental explants. Predicted targets of these differentially abundant microRNAs were linked to neurodevelopment and the placenta. The present study provides further evidence that the diseased placenta may release factors that damage cortical cells and suggests the possibility of targeted antioxidant treatment of the placenta to prevent neurodevelopmental disorders.

## Introduction

Prenatal development represents a critical period of neurodevelopment and is of importance to the long-term health of the offspring [1]. The placenta is thought to play a key role in brain development and in foetal programming of neurological disease [2–4], but the mechanism is still unclear. Preeclampsia, affecting 2–8% of pregnancies worldwide [5], is characterized by chronic gestational hypertension and significant proteinuria, along with maternal organ dysfunction or intrauterine growth restriction [6]. Not only does it increase maternal and foetal mortality, preeclampsia has long-term health implications for both mother and child, including an increased risk of schizophrenia [7–9] and autism spectrum disorders [10,11] in the offspring. In fact, according to a recent clinical meta-analysis [11], offspring exposed to preeclampsia had a 32% higher risk of developing autism spectrum disorders compared with offspring from pregnancies not complicated by preeclampsia. While aspirin is recommended as prophylaxis specifically in high-risk women [12,13], the most effective treatment is delivery of foetus and placenta [5], pointing towards a key role of the placenta in the disease. In fact, poor placentation, specifically abnormal remodelling of spiral arteries, thereby creating an ischaemic placenta, has been identified as an underlying cause of preeclampsia [14]. Studies analysing human placental tissue point towards a role of placental oxidative stress [15–22] and mitochondrial dysfunction [23–27] in preeclampsia.

We have previously observed schizophrenia-related neurodevelopmental abnormalities in adolescent rats exposed to gestational hypoxia, which were prevented when oxidative stress in the placenta was reduced [28]. Secretions from the hypoxic placenta and in the foetal blood, applied to cortical neurons, altered neuronal characteristics in a similar manner to the changes observed in the offspring brains, suggesting that factors released from the placenta may play a key role in mediating the effects of gestational hypoxia on neurodevelopment [28,29]. Using placental tissue from patients with preeclampsia, we further explore the hypothesis that secretions from the diseased placenta can cause abnormalities in cortical neurons and astrocytes.

## Results

### Conditioned medium from preeclamptic placentae affects dendrite lengths, glutamate receptor and GABA receptor levels

**Figure 1:**
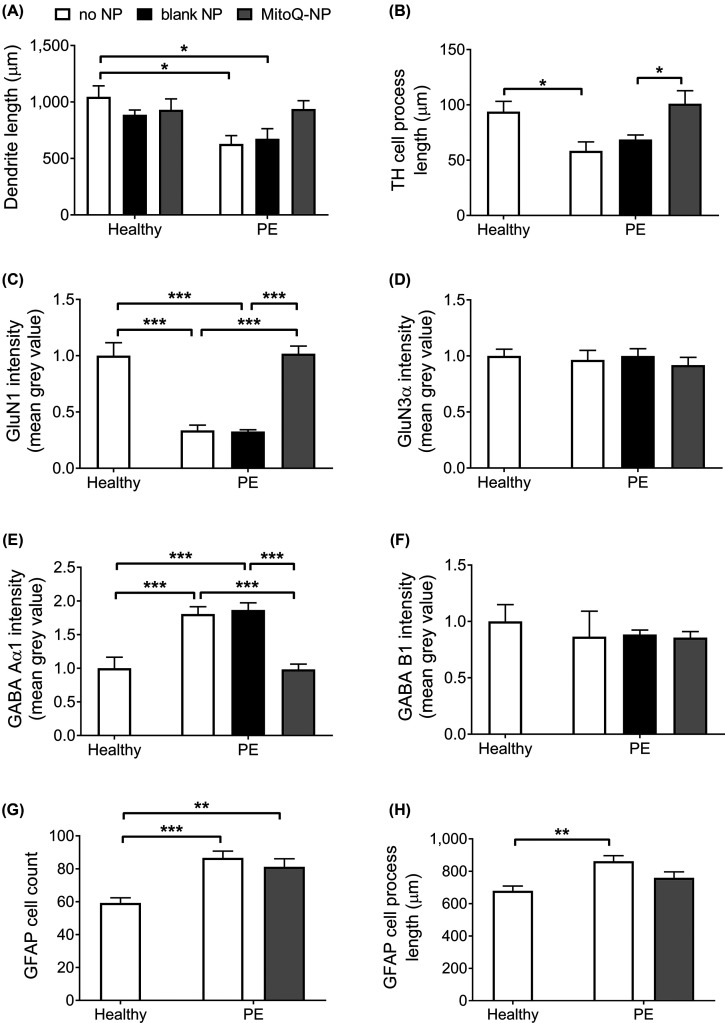
Effects of healthy and PE medium on mixed cortical cultures Mixed cortical cultures were exposed to medium conditioned by healthy (*n*=6) or preeclamptic (*n*=6) placental explants that had been pre-treated with no NPs, blank-NPs or MitoQ-NPs. Following a 24-h exposure, cortical cultures were assessed for neuronal dendrite lengths (**A**), process lengths of TH^+^ cells (**B**), levels of glutamate receptor subunits GluN1 (**C**) and GluN3α (**D**), levels of GABA receptor subunits GABA Aα1 (**E**) and GABA B1 (**F**), along with astrocyte count (**G**) and astrocyte process lengths (**H**). Data are presented as means + S.E.M.; *P<0.05, **P<0.01, ***P<0.001.

Mixed cortical cultures were exposed to culture medium conditioned by preeclamptic (PE) placental explants (PE medium) or by non-diseased explants (healthy medium) (Figure 1 and Supplementary Figure S1). PE medium applied to cortical cultures significantly reduced mean dendrite length (Figure 1A) as well as process lengths of tyrosine hydroxylase-positive (TH^+^) neurons (Figure 1B) compared with healthy medium. Levels of glutamate receptor subunit GluN1 were significantly reduced following exposure to PE medium (Figure 1C). In contrast, glutamate receptor subunit GluN3α intensity was not altered (Figure 1D). Cortical cultures exposed to PE medium showed an increase in the levels of γ-aminobutyric acid (GABA) receptor subunit GABA Aα1 (Figure 1E), while levels of subunit GABA B1 were unchanged (Figure 1F). Astrocyte numbers (Figure 1G) and process lengths (Figure 1H) were significantly increased in cortical cultures following exposure to PE medium.

Next, we queried if oxidative stress within the preeclamptic placenta could contribute to the effects of conditioned medium on cortical cultures. A previous study indicated that antioxidant MitoQ, as a nanoparticle formulation (MitoQ-NP), was able to enter placental cells, reduce oxidative stress and prevent damaging placental secretions *in vivo* [28]. We tested here if MitoQ-NP application could also prevent damaging secretions when applied to preeclamptic placental tissue *ex vivo*. PE placentae were exposed to MitoQ-NPs at the start of the *ex vivo* period. As a control, blank nanoparticles (blank-NPs) without MitoQ were used. Treatment of placental explants with MitoQ-NPs did not affect dendrite lengths of neurons exposed to healthy medium (Figure 1A). In contrast, MitoQ-NP treatment rescued the effects of PE medium on dendrite lengths (Figure 1A). Furthermore, application of MitoQ-NPs to preeclamptic placental tissue rescued the reduction in TH^+^ process lengths (Figure 1B), GluN1 levels (Figure 1C) and GABA Aα1 levels (Figure 1E) following exposure to PE medium. In addition, MitoQ-NPs partially reduced the effect of PE medium on astrocyte processes lengths so that there was no significant difference between cultures exposed to healthy or PE medium, following MitoQ-NP treatment (Figure 1H). No effect of MitoQ-NP treatment was observed on levels of GluN3α (Figure 1D) or GABA B1 (Figure 1F) or on astrocyte cell count (Figure 1G) in cultures exposed to PE medium. Application of blank NPs to placental explants did not affect dendrite lengths, TH^+^ process lengths, GluN1 levels, GluN3α levels, GABA Aα1 levels or GABA B1 levels of cortical cultures exposed to PE medium (Figure 1A–F).

Taken together, culture medium conditioned by preeclamptic placental tissue significantly altered neuronal process lengths, glutamate and GABA subunit levels as well as astrocyte numbers and process lengths. Treatment of the placental tissue with MitoQ-NPs successfully prevented several of these changes.

### Glutamate receptor inhibition prevented effects of PE medium on neuronal process lengths

**Figure 2:**
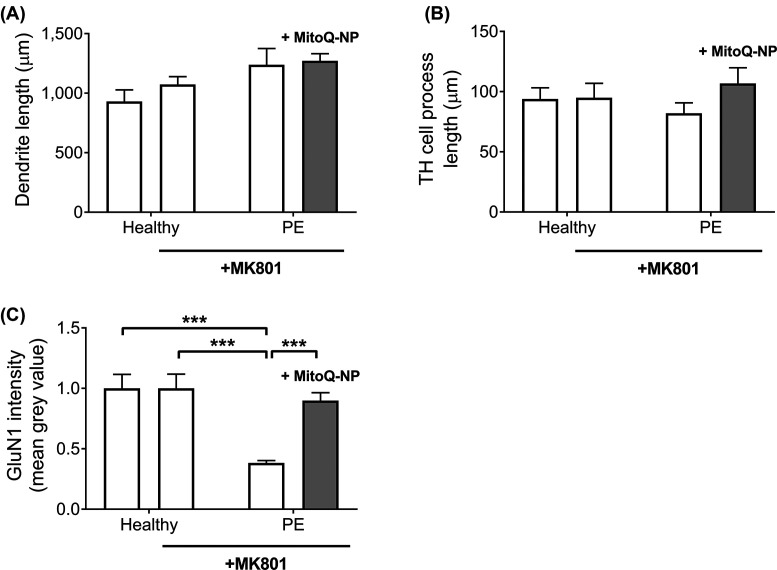
Role of glutamate inhibition in mediating effects of PE medium on mixed cortical cultures Mixed cortical cultures were incubated with NMDA receptor antagonist MK801 prior to exposure to culture medium conditioned by healthy (*n*=6) or preeclamptic (*n*=6) placental explants that had been treated with no NPs or with MitoQ-NPs. Lengths of neuronal dendrites (**A**), lengths of TH^+^ cell processes (**B**) and levels of glutamate receptor subunit GluN1 (**C**) were measured in the cortical cultures (data are presented as means + S.E.M.; ***P<0.001).

We previously identified glutamate as one of the factors that may mediate adverse effects of culture medium conditioned by a model placental barrier, exposed to hypoxia, on cortical cultures [29]. We therefore investigated if glutamate could also play a role in mediating the damaging effects of PE medium on neurons. Cortical cultures were pre-treated with MK801, a non-competitive antagonist of *N*-methyl-d-aspartate (NMDA) receptors, before incubation with healthy medium or PE medium (Figure 2 and Supplementary Figure S1). MK801 pre-treatment had no effect on dendrite lengths (Figure 2A), TH^+^ process lengths (Figure 2B) or GluN1 subunit levels (Figure 2C) in cultures exposed to healthy medium. In contrast with non-pretreated cortical cultures, which showed a shortening of dendrites following exposure to PE medium (Figure 1A), exposure of MK801-pre-treated cortical cultures with PE medium led to a non-significant increase in dendrite lengths (Figure 2A). This increase was still observed when cortical cultures were incubated with PE medium from preeclamptic explants that had been treated with MitoQ-NPs (Figure 2A). While incubation of non-pretreated cortical cultures with PE medium led to a reduction in TH^+^ process lengths (Figure 1B), exposure of MK801-pre-treated cultures to PE medium, with or without MitoQ-NP treatment, did not affect TH^+^ process lengths (Figure 2B). In contrast, as observed in non-pretreated cultures (Figure 1C), levels of GluN1 subunits were significantly reduced when MK801-pre-treated cultures were exposed to PE medium compared with healthy medium (Figure 2C). Application of MitoQ-NPs prevented this reduction in GluN1 intensity in cultures exposed to PE medium (Figure 2C). To summarize, pre-treatment of cortical cultures with a glutamate receptor inhibitor prevented the effects of PE medium on process lengths but not on GluN1 levels.

### Indirect exposure of neuron-only cultures via astrocytes replicated effects of direct exposure of mixed cultures to PE medium

**Figure 3:**
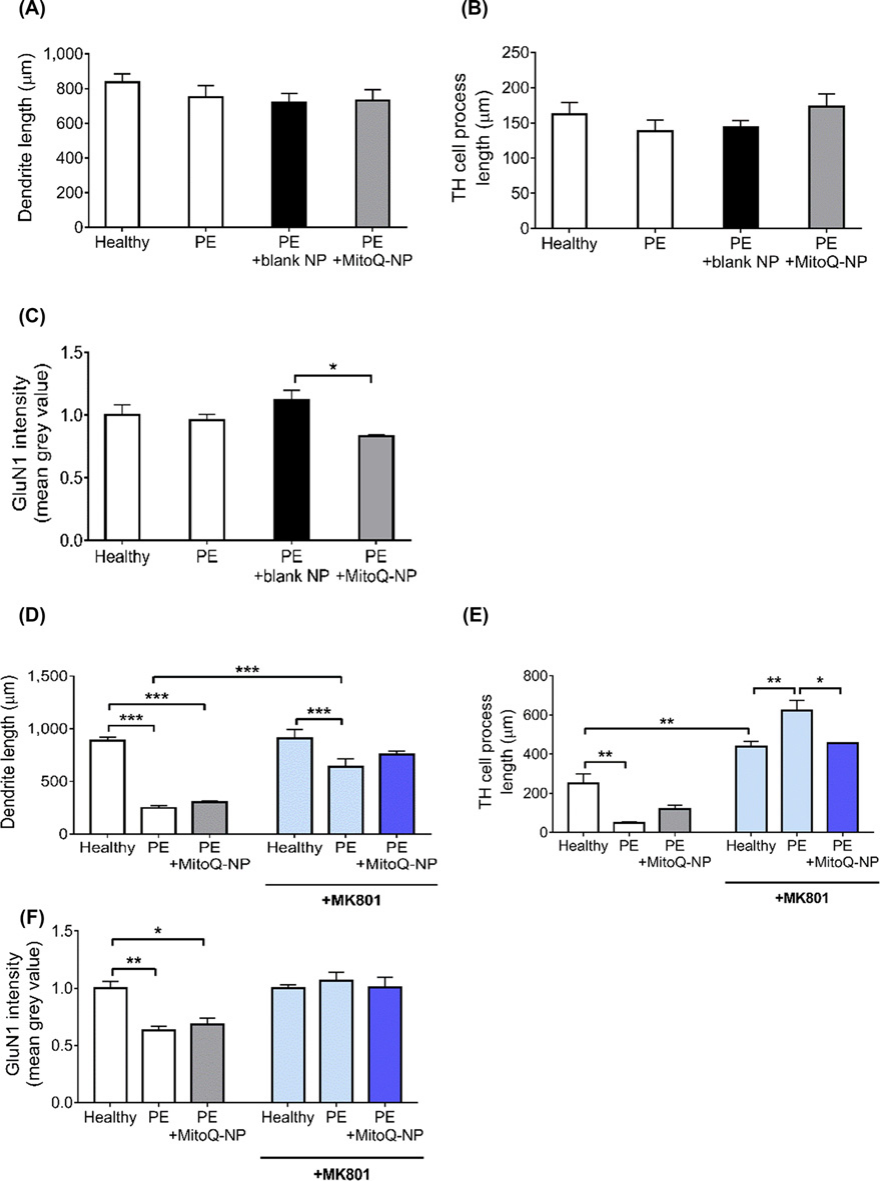
Role of astrocytes in mediating effects of PE medium on neurons (**A–C**) Neuron-only cortical cultures were exposed directly to medium conditioned by healthy or preeclamptic placental explants, which had been pre-treated with no NPs, blank-NPs or MitoQ-NPs. (**D–F**) In a second experiment, neuron-only cultures were exposed to medium collected from astrocyte-only cortical cultures, which had previously been exposed to the placenta conditioned medium, and some neuronal cultures were additionally pre-treated with NMDA receptor antagonist MK801. Neuronal dendrite lengths (A,D), TH^+^ process lengths (B,E) and glutamate receptor subunit GluN1 levels (C,F) were measured (data are presented as means + S.E.M.; *P<0.05, **P<0.01, ***P<0.001).

A recent study suggested that systemically injected lipopolysaccharide can alter the hippocampal cytokine microenvironment by interacting with astrocytes at the blood–brain barrier rather than passing through to affect the hippocampus directly [30]. Therefore, the potential role of astrocytes in mediating the damaging effects of PE medium on neurons was explored. First, we investigated if astrocytes needed to be present in order for PE medium application to affect neurons in cortical cultures. To achieve this, neuron-only cultures that did not contain any astrocytes or other glia were exposed to PE medium (Figure 3A–C and Supplementary Figure S2). In contrast with mixed cultures, where dendrite shortening was observed following application of PE medium (Figure 1A), in neuron-only cultures PE medium had no effect on dendrite lengths (Figure 3A). PE medium collected following treatment with blank-NPs or MitoQ-NPs did not alter dendrite lengths of neuron-only cultures (Figure 3A). While incubation of mixed cultures with PE medium led to a shortening of TH^+^ processes (Figure 1B), application of PE medium to neuron-only cultures did not affect TH^+^ process lengths (Figure 3B). PE medium from blank-NP- or MitoQ-NP-treated explants also did not affect TH^+^ process lengths in neuron-only cultures (Figure 3B). Compared with mixed cultures, where incubation with PE medium led to a reduction in GluN1 levels (Figure 1C), in neuron-only cultures levels of GluN1 subunits were not significantly different between neurons exposed to healthy medium or PE medium from untreated or NP-treated explants (Figure 3C). However, levels of GluN1 were significantly lower following MitoQ-NP treatment compared with blank-NP treatment (Figure 3C). Taken together, the changes in process lengths and levels of glutamate receptor subunits observed in mixed cortical cultures, following exposure to PE medium, were not replicated in neuron-only cultures, suggesting that the presence of non-neuronal cells was indeed required for PE medium to affect neurons in culture.

We next queried if astrocytes mediated the effects of PE medium on neurons via release of signalling molecules. Astrocyte-only cultures, devoid of neurons, were exposed to PE medium for 24 h to collect conditioned medium (PE-astrocyte medium). PE-astrocyte medium was then applied to neuron-only cultures to assess if this replicated the effects of direct application of PE medium to mixed cortical cultures (Figure 3D–F and Supplementary Figure S2). As observed after exposure of mixed cultures to PE medium directly (Figure 1A), dendrite lengths were significantly reduced in neuron-only cultures following exposure to PE-astrocyte medium (Figure 3D). Treatment of the preeclamptic explants with MitoQ-NPs did not ameliorate this effect. In line with the observed effects of PE medium on mixed cortical cultures (Figure 1B), TH^+^ process lengths in neuron-only cultures were also significantly reduced following exposure to PE-astrocyte medium (Figure 3E). This reduction was slightly less pronounced when preeclamptic explants were treated with MitoQ-NPs. Like mixed cultures exposed to PE medium directly (Figure 1C), neuron-only cultures showed significantly decreased GluN1 subunit levels following exposure to PE-astrocyte medium, independent of whether preeclamptic explants had been treated with MitoQ-NPs or not (Figure 3F). These results indicate that signalling via astrocytes is required for neurons to be affected by PE medium.

Based on the results described above, functioning glutamate receptors appear to be required for some of the observed effects of PE medium on neurons. Therefore, we investigated if glutamate signalling from astrocytes to neurons may be involved in mediating the effects of PE medium on neurons. When glutamate signalling in neuron-only cultures was inhibited by MK801, exposure to PE-astrocyte medium still significantly reduced dendrite lengths but to a lesser extent than without MK801 pre-treatment. MitoQ-NP application did not alter dendrite lengths following MK801 pre-treatment (Figure 3D). TH^+^ process lengths were significantly increased following exposure to healthy astrocyte medium and application of PE-astrocyte medium led to a significant additional increase in process lengths, when neuron-only cultures were pre-treated with MK801. These effects were prevented by MitoQ-NP treatment of the placental tissue (Figure 3E). Inhibition of glutamate receptors in neuron-only cultures by MK801 pre-treatment prevented the effect of PE-astrocyte medium on GluN1 levels in neuron-only cultures. MitoQ-NP treatment had no effect on GluN1 levels in pre-treated cultures. It appears that glutamate signalling from astrocytes to neurons may well be one of the underlying mechanisms mediating the effects of PE medium on neurons.

### Indirect exposure of astrocyte-only cultures via neurons partially replicated effects of direct exposure of mixed cultures to PE medium

**Figure 4:**
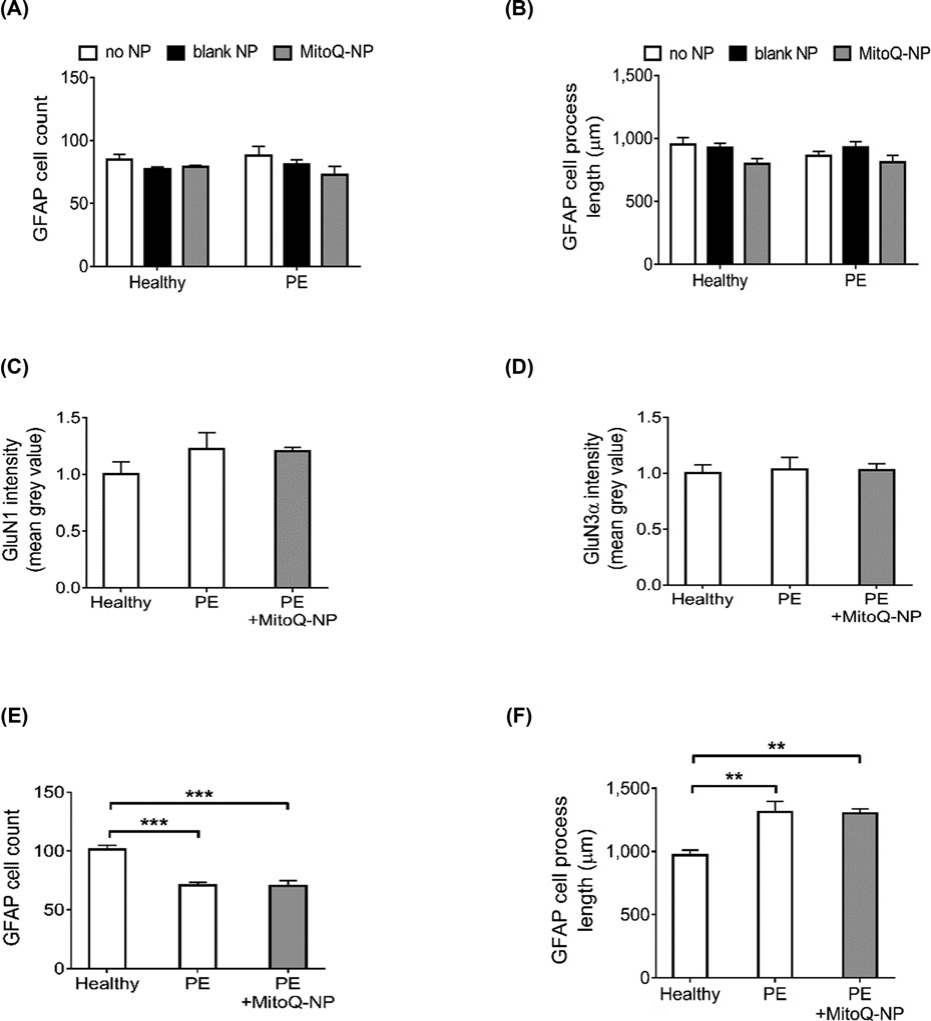
Role of neurons in mediating effects of PE medium on astrocytes (**A** and **B**) Astrocyte-only cortical cultures were exposed directly to medium conditioned by healthy or preeclamptic placental explants, which had been pre-treated with no NPs, blank-NPs or MitoQ-NPs. (**C–F**) In a second experiment, astrocyte-only cultures were exposed to medium collected from neuron-only cortical cultures, which had previously been exposed to the placenta conditioned medium. Astrocyte cultures were assessed with regard to cell count (A and E), process lengths (B and F) along with levels of glutamate receptor subunits GluN1 (C) and GluN3α (D) (data are presented as means + S.E.M.; **P<0.01, ***P<0.001).

We further investigated if signalling in the opposite direction, from neurons to astrocytes, could have an additional role in mediating the effects of PE medium on cortical cultures. As above, we first assessed if the presence of neurons was required for PE medium to cause changes in astrocytes. PE medium was applied directly to astrocyte-only cultures that did not contain any neurons (Figure 4A–D). In contrast with mixed cultures, where increases in astrocyte cell count and process length were observed following exposure to PE medium (Figure 1G,H), astrocyte-only cultures did not show any changes in astrocyte cell count (Figure 4A) or process length (Figure 4B) after application of PE medium. Exposure of astrocyte-only cultures to healthy or PE media conditioned by blank-NP- or MitoQ-NP-treated explants did not affect cell count or process lengths. Levels of glutamate receptor subunits GluN1 (Figure 4C) and GluN3α (Figure 4D) were unaffected in astrocyte-only cultures treated with PE medium from untreated or MitoQ-NP-treated explants. To summarize, exposure of astrocyte-only cultures to PE medium did not reproduce the effects on astrocytes observed in mixed cultures exposed to PE medium, suggesting that the presence of neurons may be required for PE medium to exert its effects on astrocytes.

To investigate if signalling from neurons to astrocytes plays a role in mediating the effects of PE medium on astrocytes, astrocyte-only cultures were exposed to culture medium conditioned by neuron-only cultures that had been incubated with PE medium (PE-neuron medium) (Figure 4E,F and Supplementary Figure S3). In contrast with mixed cortical cultures that showed an increase in astrocyte count following direct exposure to PE medium (Figure 1G), astrocyte-only cultures incubated with PE-neuron medium had significantly reduced numbers of astrocytes (Figure 4E). Prior MitoQ-NP treatment of preeclamptic explants did not ameliorate this effect. As was observed in mixed cultures following exposure to PE medium, process lengths of astrocyte-only cultures were significantly increased after incubation with PE-neuron medium (Figure 4F). MitoQ-NP treatment did not affect astrocyte process length. Thus, the observed increase in astrocyte process length following application of PE medium to cortical cultures may involve signalling from neurons to astrocytes.

Taken together, the results suggest that the presence of neurons may be required for PE medium to exert its effect on astrocyte process length but not astrocyte number.

### Altered microRNA secretion profile from PE explants

The above results suggest that PE medium may contain factors that damage neurons and astrocytes in culture. Any attempt to identify with certainty the factors released from the placenta that may affect cortical cultures is outside the scope of this study. Instead, we investigated if the content of the PE medium is altered compared with medium conditioned by healthy placentae, focussing on two relevant classes of molecules.

**Figure 5:**
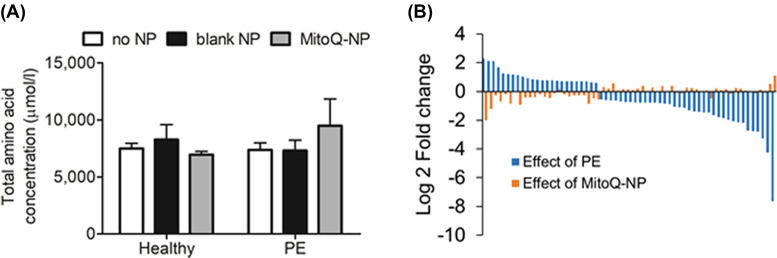
Characterization of PE conditioned medium (**A**) Total levels of proteinogenic amino acids (µmol/l ± S.E.M.) were measured in culture medium conditioned by healthy (*n*=3) or preeclamptic (*n*=3) placental explants, following pre-treatment with no NPs, blank-NPs or MitoQ-NPs. (**B**) Log 2 fold change values are shown for those microRNAs that were significantly altered in PE medium (*n*=4) compared with healthy medium (*n*=4) (blue). Log fold changes of those same microRNAs in medium conditioned by preeclamptic placental explants treated with MitoQ-NPs (*n*=3), compared with untreated PE medium, are shown in orange.

Firstly, the results thus far implicated glutamate signalling in mediating the damaging effects of PE medium on neurons. This glutamate signalling may occur within cortical cultures (i.e. from astrocytes to neurons) or may originate directly from the placenta. Glutamate and other amino acids play an important role in foetal development and growth [31]. Therefore, we investigated if PE medium might affect cortical cultures through altered levels of amino acids compared with healthy medium. Biochemical analysis of proteinogenic amino acids revealed no significant differences in total or individual levels of amino acids between PE medium and healthy medium (Figure 5A and Table 1), suggesting that altered secretion of amino acids from the preeclamptic placenta is not a mechanism by which PE medium affects cortical cells.

**Table 1:** Amino acid concentration in μmol/l (±S.E.M.) in medium conditioned by healthy (*n*=3) or preeclamptic placentas (*n*=3), after exposure to no NPs, blank-NPs or MitoQ-NPs

	Healthy			PE		
Amino acid	No NP	Blank-NP	MitoQ-NP	No NP	Blank-NP	MitoQ-NP
Ala	79.27 (45.02)	90.93 (64.43)	54.77 (24.70)	79.70 (52.22)	64.07 (42.47)	251.0 (228.84)
Arg	357.0 (5.483)	335.7 (33.79)	331.8 (19.46)	336.6 (6.552)	332.7 (17.84)	371.1 (26.71)
Asn	16.63 (16.63)	21.33 (21.33)	7.833 (7.833)	15.07 (15.07)	11.70 (11.70)	94.13 (94.13)
Asp	0 (0)	0 (0)	0 (0)	0 (0)	0 (0)	67.17 (67.17)
Cys	58.63 (7.636)	58.10 (2.001)	62.60 (7.614)	67.57 (4.927)	69.83 (4.190)	66.27 (4.983)
Gln	1509 (396.8)	1411 (275.0)	1358 (318.2)	1293 (436.2)	1273 (442.3)	1464 (477.7)
Glu	64.73 (53.55)	67.90 (67.90)	36.10 (30.62)	41.00 (24.97)	62.97 (50.80)	201.4 (188.5)
Gly	409.4 (34.20)	384.3 (51.72)	356.8 (24.70)	398.9 (30.74)	393.3 (52.11)	585.4 (223.0)
His	153.3 (12.70)	143.4 (21.24)	142.9 (15.69)	127.8 (7.503)	139.2 (10.12)	185.2 (59.89)
Ile	702.4 (35.45)	629.6 (17.69)	647.5 (28.17)	683.4 (48.12)	682.9 (46.88)	770.0 (59.13)
Leu	554.4 (192.9)	674.5 (23.68)	671.0 (24.70)	725.4 (30.02)	720.2 (53.58)	908.5 (177.8)
Lys	736.7 (17.91)	674.5 (36.12)	675.6 (26.43)	726.3 (15.76)	721.9 (61.11)	890.9 (170.8)
Met	171.6 (7.270)	154.1 (4.206)	152.5 (7.878)	162.5 (10.52)	175.0 (13.83)	208.4 (40.77)
Phe	377.8 (14.26)	344.5 (15.59)	346.3 (14.85)	386.8 (9.103)	390.2 (33.93)	467.3 (79.52)
Pro	86.00 (17.56)	87.20 (34.33)	72.50 (12.85)	146.1 (66.25)	100.4 (33.79)	252.7 (188.6)
Ser	377.4 (9.481)	346.9 (23.40)	342.9 (13.37)	369.1 (13.16)	358.1 (31.58)	514.3 (151.0)
Thr	740.6 (23.73)	671.67 (27.48)	679.6 (27.09)	711.6 27.48)	716.3 (53.71)	829.0 (106.1)
Trp	0 (0)	0 (0)	0 (0)	0 (0)	0 (0)	23.73 (23.73)
Tyr	366.2 (10.53)	330.2 (14.00)	336.4 (13.60)	364.8 (12.40)	355.7 (26.76)	433.0 (60.29)
Val	733.8 (27.62)	673.4 (23.32)	671.8 (26.17)	734.27 (27.35)	741.7 (56.19)	893.6 (153.5)

Secondly, we analysed levels of microRNAs, post-transcriptional regulators with relevance to neurodevelopment [32] and preeclampsia [33]. In a previous study, we observed differential secretion of microRNAs from the rodent placenta in response to placental oxidative stress [28]. Therefore, we hypothesized that microRNA secretion from the preeclamptic placenta might also be altered. A NanoString platform analysis revealed 60 microRNAs with significantly different abundance in PE medium compared with healthy medium (*P*<0.05; Figure 5B). Predicted targets of the significant microRNAs are listed in Supplementary Table S1. Predicted targets were significantly enriched for biological processes linked to nervous system development (Table 2) and for genes associated with the brain (*P*=7.30E-25) and placenta (*P*=2.80E-12). Pathways enriched among the predicted targets of significant microRNAs included several signalling pathways as well as pathways with specific relevance to neurons, such as axon guidance (Table 3). MitoQ-NP treatment of the preeclamptic explants partially prevented the changes in microRNA abundance (Figure 5B). Specifically, seven of the microRNAs found to be differentially abundant in PE medium were also significantly altered in their abundance following MitoQ-NP, but not blank-NP, treatment and levels were partially returned to control levels (Table 4).

**Table 2:** Enriched biological processes

Biological process	Count	Fold enrichment	*P* value
Sensory perception of chemical stimulus	3	<0.2	3.20E-23
Developmental process	468	1.53	1.99E-17
Sensory perception of smell	1	<0.2	1.17E-16
Intracellular signal transduction	262	1.67	4.44E-13
Sensory perception	40	0.38	3.47E-11
Signal transduction	518	1.37	4.18E-11
MAPK cascade	108	2.16	1.16E-10
Cell communication	565	1.34	1.88E-10
Cell cycle	226	1.59	5.55E-09
Nervous system development	173	1.64	1.42E-07
System development	248	1.47	5.14E-07
Cellular process	1461	1.13	8.56E-07
Death	118	1.74	3.21E-06
Cell death	118	1.74	3.21E-06

Results of gene ontology analysis of predicted targets of differentially abundant microRNAs in PE placenta conditioned medium compared with healthy placental conditioned medium are listed. *P* values have been corrected for multiple comparisons using FDR.

**Table 3:** Top 15 enriched pathways

KEGG pathway	Count	Fold enrichment	*P* value
MAPK signalling pathway	106	2.40	1.11E-17
Ras signalling pathway	89	2.27	5.58E-13
Pathways in cancer	127	1.86	1.51E-11
Neurotrophin signalling pathway	55	2.64	8.13E-11
Rap1 signalling pathway	79	2.17	1.58E-10
PI3K-Akt signalling pathway	112	1.87	1.89E-10
Melanoma	37	3.00	4.36E-09
Axon guidance	53	2.41	8.11E-09
ErbB signalling pathway	41	2.72	1.44E-08
FoxO signalling pathway	54	2.32	1.98E-08
Renal cell carcinoma	33	2.93	7.95E-08
T-cell receptor signalling pathway	44	2.46	9.83E-08
Signalling pathways regulating pluripotency of stem cells	54	2.22	9.61E-08
Regulation of actin cytoskeleton	71	1.94	2.24E-07
Insulin signalling pathway	52	2.17	4.19E-07

Results of KEGG pathway analysis of predicted targets of differentially abundant microRNAs in PE placenta conditioned medium compared with healthy placental conditioned medium are listed. *P* values have been corrected for multiple comparisons using Benjamini–Hochberg method.

**Table 4:** microRNAs significantly affected in preeclampsia and by MitoQ-NP treatment

microRNA	Known relevant functions
**Up-regulated in PE**	
hsa-miR-561-5p	Regulates 11β-HSD1, which is highly expressed in liver, adipose tissue and CNS [96]
	Associated with Parkinson’s disease [97]
hsa-miR-548ai+hsa-miR-570-5p	miR-570 associated with autism [98] and congenital heart disease [99]
	miR-570 regulates cytochrome P450 [100]
hsa-miR-196b-5p	Associated with ectopic pregnancy [101]
	Associated with endometriosis [102]
hsa-miR-2117	None
hsa-miR-3065-3p	None
**Down-regulated in PE**	
hsa-miR-596	None
hsa-miR-451a	Associated with type 2 diabetes [103]

Listed are those microRNAs that were found to be up- or down-regulated in culture medium conditioned by preeclamptic placental explants, compared with healthy explants, and that were also affected in the opposite direction by treatment of the placenta with MitoQ-NPs but not blank-NPs.

## Discussion

### Conditioned medium from preeclamptic placentae affects cortical cultures

The placenta is thought to play a key role in foetal programming of neurological disorders yet the process of how the placenta can affect neurodevelopment as a result of pregnancy complications remains poorly understood [3]. The present study investigated if secreted molecules from the diseased placenta could affect neurons and astrocytes *in vitro* by exposing cortical cultures to culture medium conditioned by preeclamptic placentae. Exposure of cortical cells to PE medium replicated some of the core pathological changes observed in diseases associated with preeclampsia, such as schizophrenia [7,9] and autism spectrum disorders [10]. Recent studies have implicated neural circuit dysfunction in the aetiology of neurodevelopmental disorders [34], including a potential imbalance of excitatory and inhibitory inputs [35]. In cortical cultures exposed to PE medium, we observed reduced levels of GluN1 subunits and increased levels of GABA Aα1, while levels of subunits GluN3α and GABA B1 were unaffected. Dysregulation of NMDA receptors [36–39] and GABA receptors [40] is hypothesized to represent a key contributor to schizophrenia aetiology and has been observed in post-mortem brain tissue of patients with schizophrenia or autism, as well as in animal models. The subunit composition of NMDA receptors changes during development [41–45]. Essential subunit GluN1 forms heteromeric complexes with various combinations of regulatory subunits, consisting of GluN2α-δ and GluN3α/β. These modulatory subunits define the NMDA receptor properties, including calcium permeability [46], induction of synaptic plasticity [47,48] and decay kinetics [44,49]. A developmental switch has been observed in early postnatal development from GluN2α to GluN2β [41–44] as well as from GluN3α to GluN3β [45,46], leading to altered NMDA characteristics. While we might hypothesize that the observed reduction in GluN1 subunit levels in cortical cultures, following application of PE medium, correlates with a reduction in total NMDA receptor levels, a further exploration of GluN2 and GluN3 subunit levels would provide insight into potential modulatory effects of PE on NMDA receptors that may impact on their function and thereby contribute to altered neurodevelopment. Subunit composition is also critical for GABA A receptors. These receptors are thought to play an important role in neuronal proliferation, migration and synapse formation during development [50,51]. They can be located at the synapse or extrasynaptically, where they generate phasic or tonic inhibition, respectively [52,53]. As phasic and tonic GABA A receptors tend to differ in their compositions of α, β, γ/δ subunits [52,53], electrophysiological analysis of the effects of PE conditioned medium on cortical cultures would be of interest in order to delineate the changes in different GABA A receptor subunits and the concomitant effects on phasic versus tonic inhibition.

Further observations in cortical cultures exposed to PE medium were reductions in dendrite lengths as well as a shortening of TH^+^ process lengths. In fact, cortical dendrite dysgenesis [36,54] has been observed in temporal, frontal and occipital cortices of autism and schizophrenia patients, along with changes in the dopamine system [37,55]. Lastly, our observation that astrocyte number and process lengths were reduced, is in agreement with the hypothesis that altered astrocyte number or function may represent critical changes in autism and schizophrenia brains [56–58].

The finding that application of PE medium, presumably containing molecules released from the placenta, produced changes in cortical cultures similar to those observed in brains of patients with relevant neurodevelopmental disorders, points towards a role of secreted placental factors in neurodevelopmental disease.

As oxidative stress in the placenta has been strongly implicated in preeclampsia [15–22], we investigated the possible role of placental oxidative stress in mediating the release of placental factors that damage cortical cells. Treatment of preeclamptic placental explants with nanoparticle-bound MitoQ, an antioxidant that is specifically targeted to mitochondria [59] and that has previously been shown to reduce oxidative stress in the placenta *in vivo* [28], prevented all neuron-related effects of the PE medium but did not significantly affect astrocyte number or astrocyte process lengths. While the effects of MitoQ-NP treatment on oxidative stress in the preeclamptic placenta need to be further characterized – including the testing of different doses – the presented results suggest that placental oxidative stress could have a mechanistic role in the observed neuronal changes.

### Signalling between astrocytes and neurons is required for the effects of PE medium on cortical cultures

While the data suggest that as yet unidentified molecules from the preeclamptic placenta could affect cortical cells in culture, their mechanism of action is unknown. To investigate the potential involvement of astrocytes in mediating the effects of PE medium on neurons, we incubated neuron-only cultures, without the presence of astrocytes, with PE medium. We observed none of the effects on dendrite lengths, glutamate or GABA receptor levels that were seen when PE medium was applied to mixed cultures that contained both neurons and astrocytes. Conversely, incubation of astrocyte-only cultures with PE medium did not replicate the effects seen following PE medium application to mixed cultures. This indicates that the presence of both astrocytes and neurons is required for PE medium to affect cortical cells, thus signalling between these two cell types may play an important role. Bidirectional cross-talk between astrocytes is clearly important during neurodevelopment. Astrocytes are involved in neuronal migration and survival, neurite extension and synapse maturation, thereby supporting correct development of neural circuits [60]. Conversely, the presence of neurons induces typical stellate morphology in astrocytes and astrocytic gene expression may be regulated by neuronal activity [61]. Furthermore, astrocytes within the blood–brain barrier have been shown to mediate signalling of circulating factors, such as lipopolysaccharide, to neurons, without these factors passing through the blood–brain barrier [30].

Exposure of astrocyte-only cultures to PE medium and subsequent application of this conditioned medium to neuron-only cultures reproduced the effects of direct PE medium application to mixed cultures. Furthermore, exposure of astrocyte-only cultures to medium conditioned by neuron-only cultures that had been exposed to PE medium replicated the effect on astrocyte process length, but not astrocyte number. These findings further support the hypothesis that bidirectional communication between astrocytes and neurons plays a role in mediating the effects of PE medium on cortical cells. MitoQ-NP treatment of preeclamptic placentae did not rescue any of the observed effects of indirect exposure of cortical cells to PE medium via neurons or astrocytes. This stands in contrast with the preventative effect of MitoQ-NP treatment that was observed when PE medium was applied to mixed cultures directly. While the reason for this observation remains unknown, it is likely that the mechanism by which MitoQ-NP treatment may prevent harmful secretion of molecules from the preeclamptic placenta is not as simple as reversing the secretion profile of the placenta back to a ‘healthy’ profile. The preeclamptic placenta may release ‘damaging’ factors that cause abnormalities in neurons and astrocytes. In contrast, following MitoQ-NP treatment, the placenta may alter its secretions to send out ‘supportive’ signals. It is possible that the ‘supportive’ signal from the placenta following MitoQ-NP application has no effect on astrocyte-only cultures or on astrocyte signalling to neurons. Indeed, as described above, MitoQ-NP treatment had no effect on astrocytic abnormalities following direct application of PE medium to cortical cultures. Therefore, this ‘supportive’ signal acts either on neurons directly (which is only possible when applied directly to mixed cortical cultures) or on other non-astrocytic glial cells only present in the mixed cultures. Indeed, microglial cells play a critical role in the developing brain, are affected in many neurological disorders and microglial priming, the disruption of microglia due to prenatal insults, can increase the vulnerability to psychiatric disorders in later life [62]. Microglia may therefore also be affected by PE medium and/or MitoQ-NP treatment.

To conclude, the results suggest that astrocyte–neuron signalling is required for mediating most (but not all) effects of PE medium on cortical cells.

### Glutamate signalling within cortical cultures may mediate the effects of PE medium on neurons

Inhibition of glutamate receptors in mixed cortical cultures rescued the effects of PE medium on neuronal process lengths, suggesting that glutamate may mediate some of the effects of PE medium on cortical cultures. As the major excitatory neurotransmitter in the brain, imbalance in glutamate signalling is a hallmark of complex neurological disorders, including schizophrenia and autism [36–39]. Bidirectional signalling via glutamate between neurons and astrocytes plays an important role in synaptic function, as astrocytes can modulate synaptic transmission via glutamate release at the tripartite synapse [63], glutamate clearance from synaptic cleft [64–66] and control of glutamate diffusion in the extracellular space [67]. As application of PE medium caused changes in astrocyte number, altered management of extracellular glutamate by astrocytes could potentially play a role in mediating the effects of PE medium on neurons.

Glutamate receptor inhibition in neuron-only cultures exposed to astrocyte-PE medium, rescued neuronal process lengths and GluN1 levels. In fact, process lengths were increased following application of healthy medium to neurons pre-treated with glutamate receptor inhibitor. Taken together with the mixed culture results, glutamate receptor inhibition has different effects, depending on whether glutamate signalling was inhibited in general or specifically with regard to astrocyte-to-neuron signalling. This suggests that other factors are involved in mediating the effects of PE medium on neurons, in addition to glutamate. Previous studies have implicated other amino acids, neurotransmitters (or gliotransmitters), neuropeptides, cytokines and small signalling molecules (NO, CO) in the bidirectional neuron–astrocyte cross-talk [68,69].

### microRNAs as candidate placental signalling molecules

In the present study, we observed an altered microRNA secretion profile from the preeclamptic placenta compared with healthy controls. Predicted targets of these differentially secreted microRNAs were enriched for nervous system development, brain and placenta. MitoQ-NP application partially ameliorated the altered secretion profile, with seven microRNAs being affected both by disease status and treatment. This observation correlates with the suggestion from the cortical culture data that MitoQ-NP application in this model does not necessarily return the placental secretion profile to ‘healthy’ levels. Instead, it appears that MitoQ-NP treatment alters the secretion levels of selected microRNAs, together with other active factors, to an extent that is sufficient to prevent the damaging effects of PE medium on neurons.

microRNAs have been shown to be released from a variety of cells into the blood and other body fluids and to be taken up by recipient cells, including neurons and astrocytes, where they are able to exert their functional role of post-transcriptional regulation of gene expression [70]. Other glial cells not investigated in the present study, such as microglia, have also been shown to take up extracellular microRNAs [71]. Thus, their function could also be affected by microRNAs released from a preeclamptic placenta and could contribute to associated neurodevelopmental dysfunction. In addition to their role in neurodevelopment [32] and neuropsychiatric disorders [32,72], microRNAs are released from the placenta within exosomes into the maternal blood. They are thought to provide a feedback mechanism to the maternal system and have been assessed as potential biomarkers for preeclampsia [73–75], gestational hypertension [76] and foetal growth [77], among others. We have recently shown in a model of gestational hypoxia that the hypoxic placenta differentially secretes microRNAs while the microRNA profile in the foetal blood is also altered [28]. Levels of these differentially abundant microRNAs negatively correlated with mRNA levels of their predicted targets in the foetal brain. Moreover, predicted targets of the differentially secreted microRNAs were enriched for schizophrenia-linked copy number variants, pointing towards a potential role of microRNAs in mediating the effects of a diseased placenta on the foetal brain [28]. Whether placental microRNAs secreted into the foetal circulation reach and enter the brain is so far unknown. It has been shown, however, that exosomes carrying RNA molecules are able to cross the blood–brain barrier [78]. Therefore, it is possible that microRNAs released from the placenta may influence developmental changes in the brain.

Much of the literature has focussed on factors released from the placenta towards the maternal, rather than the foetal, circulation [79]. And indeed, many proteins that are produced by the placenta are detected in much higher levels in the maternal blood compared with cord blood, suggesting that the placenta preferentially secretes these factors towards the maternal circulation [80]. However, other factors have recently been identified that are released from the placenta towards the foetus and taken up into the foetal brain, where they regulate neurodevelopmental processes, such as serotonin [81]. This response was altered following exposure to noxious stimuli including immuno-stimulation [82]. Clearly, there is a need for a better understanding of placental factors that are released towards the foetal circulation and affect foetal development.

While the differential release of microRNAs from the PE placenta needs to be independently validated, as well as their functional role confirmed and understood on a mechanistic level, the present results demonstrated that microRNA secretion from the preeclamptic placenta is perturbed and could thus constitute a potential communication mechanism between the placenta and foetal cortical cells. It is likely that any such microRNA involvement acts in concert with other secreted factors, such as proteins and hormones, as well as molecules that cross the placenta from the maternal milieu. Comprehensive characterization of the conditioned medium will be required to fully identify the range of factors released from the placenta that may influence foetal development as a result of preeclampsia.

## Conclusion

**Figure 6:**
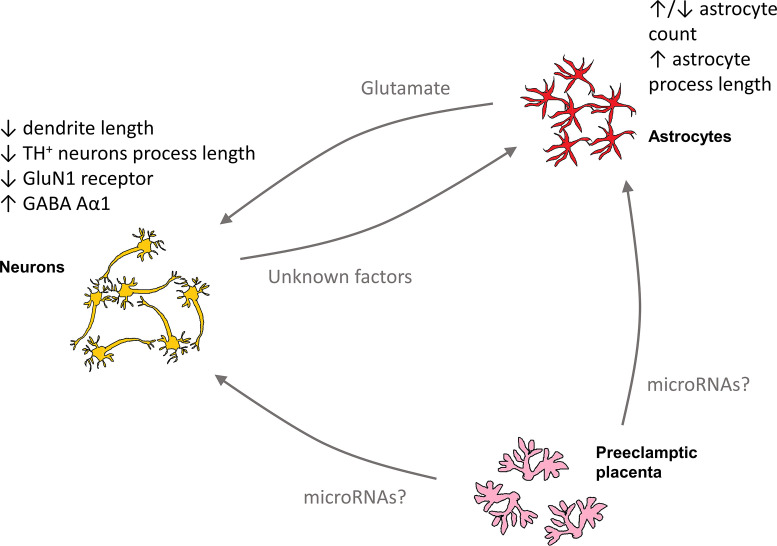
Overview of potential mechanistic effects of PE medium on cortical cultures Secreted molecules from the preeclamptic placenta, including potentially microRNAs, may have direct effects on neurons and astrocytes. Some of the effects of PE medium on cortical cultures may also be indirect via glutamate secretion from astrocytes or by secretion of unknown factors from neurons.

In the present study, we demonstrated that secretions from the PE placenta affected neurons and astrocytes in culture (Figure 6), suggesting that placental signalling to the foetal brain, potentially induced by oxidative stress in the placenta, may be important for neurodevelopment.

Analysis of what the PE placenta secretes could also provide further insight into the factors that the placenta releases into the maternal circulation, in PE. Previous studies have identified a reduction in small signalling molecules (H_2_S, CO, NO) and increases in pro-inflammatory mediators, procoagulant factors and in some cases anti-angiogenic factors released from the PE placenta [79]. Furthermore, a variety of vesicles are secreted from the syncytiotrophoblast and the composition of their cargo of proteins, RNA and microRNAs, is thought to be altered in PE [83,84]. Systemic effects of these altered circulating factors may contribute to the maternal syndrome in PE and to the possible onset of seizures, termed eclampsia. Whether placental factors released into the maternal circulation could cross the blood–brain barrier, which is thought to be more permeable in PE [85], cause neurological changes in the maternal brain and thereby increase the brain’s vulnerability to seizures is unknown. It is of relevance, however, that magnesium sulphate, which is used clinically for prevention of eclamptic seizures, is an NMDA receptor antagonist [86]. As placental pathology is central to the development of the maternal syndrome in PE, treatment of the placenta with an antioxidant might not only lead to a change in placental molecules released towards the foetus, but also towards the mother. We have previously shown in a rat model of hypoxia during pregnancy that treatment of the mother with MitoQ-NP, which rescued neurodevelopment in the foetus, also reduced hypoxia-induced oxidative stress in the maternal brain and liver [28]. No obvious side effects on the mother were observed; however, the effects of MitoQ-NP treatment on the mother require comprehensive characterization. Future studies will show if treatment of the placenta may also have beneficial implications for the mother.

Further exploration of the mechanistic link between the release of molecules from the placenta and changes in cortical cells will provide a deeper understanding of the foetal origin of neurodevelopmental diseases, as well as open up opportunities for treating of the placenta with targeted drugs to reduce the risk of neurodevelopmental disease in offspring of complicated pregnancies.

## Materials and methods

### Experimental design

**Figure 7:**
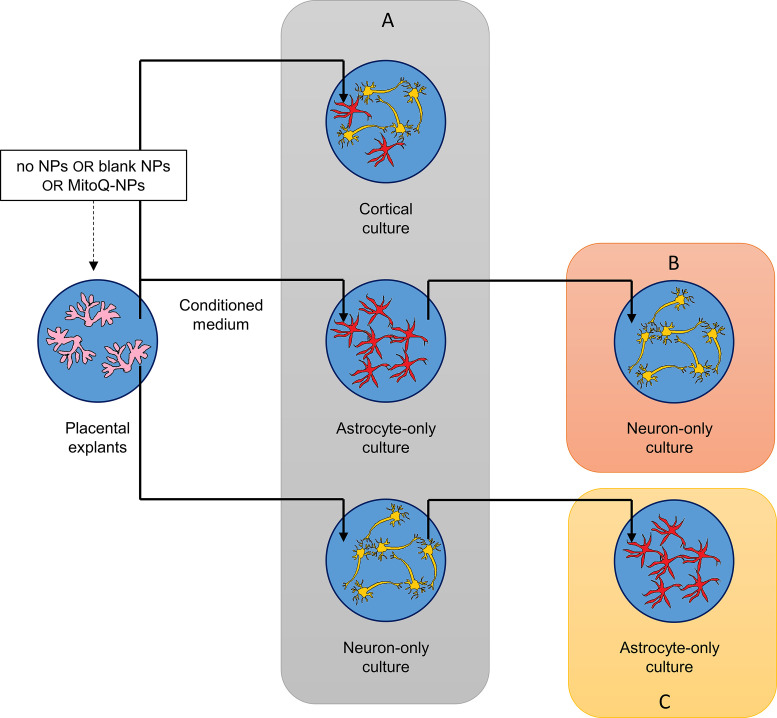
Overview of experimental setup Culture medium conditioned by healthy or preeclamptic placental explants, following exposure to no NPs, blank-NPs or MitoQ-NPs, was applied to mixed cortical cultures, astrocyte-only cortical cultures or neuron-only cortical cultures (**A**). To establish the role of astrocytes in mediating the effects of placenta-conditioned medium on cortical cultures, culture medium from the exposed astrocyte-only cultures was applied to neuron-only cultures (**B**); in order to investigate the role of neurons in mediating the effects of placenta condition medium on cortical cultures, culture medium from neuron-only cultures that had been exposed to placenta-conditioned medium was applied to astrocyte-only cultures (**C**).

Explants from preeclamptic or healthy human placentae were cultured for 24 h with addition of blank-NPs, MitoQ-NPs or without NPs. The conditioned medium was added to mixed cortical cultures, astrocyte-only cultures or neuron-only cultures for 24 h (Figure 7A), after which cultures were assessed by immunohistochemistry, as described below. In a second step, conditioned medium was collected from astrocyte-only cultures and added to neuron-only cultures for 24 h (Figure 7B). Alternatively, conditioned medium was obtained from neuron-only cultures and applied to astrocyte-only cultures (Figure 7C). This approach enabled investigation of the roles of astrocytes and neurons in mediating the effects of placenta-conditioned medium on cortical cultures.

### Tissue collection

De-identified human tissue was obtained from Southmead Hospital (Bristol, U.K.) with patients’ written informed consent and ethical approval by the NHS Health Research Authority. Mild, moderate or severe preeclampsia was diagnosed based on NICE clinical guideline 107 (Table 5). Preeclamptic placentae were delivered by induction or caesarean section. Control samples were obtained from non-diseased women following elective term caesarean delivery. Within 30 min of delivery, explants of villous tissue (∼0.5 cm^3^) were dissected from the placenta midway between chorionic and basal plates.

**Table 5:** Clinical data of collected preeclamptic placentae

	Gestation	Parity	Severity	PCR	IUGR	Co-morbidities
1*	35+5	0	Moderate	274	Unknown	n/a
2*	37+3	0	Moderate	361	No	n/a
3^*†‡^	40+8	2	Mild	204	No	n/a
4*	40+13	0	Moderate	331	No	Hypothyroid
5^*^‡^^	37	0	Severe	427	Unknown	DM1
6^*^‡^^	27+4	0	Severe	537	Yes	APKD
7^*^†‡^^	26+1	1	Severe	448	Yes	SLE nephritis, previous DVT/pulmonary embolus, baby has metabolic disorder
8^*^†^^	38+4	4	Severe	32	No	n/a

APKD, adult polycystic kidney disease; DM1, type 1 diabetes; DVT, deep vein thrombosis; IUGR, intrauterine growth restriction; PCR, protein-creatinine ratio; SLE, systemic lupus erythematosus. Conditioned media from the highlighted placentas was *applied to cortical cultures; ^†^analysed for amino acids; ^‡^analysed for microRNA.

### Conditioned medium

MitoQ (gift from Dr Michael Murphy, University of Cambridge, U.K.) [59] and γ-PGA-Phe nanoparticles (gift from Dr Mitsuru Akashi, Osaka University,Japan ) [87] were combined to produce MitoQ-NPs [28]. Following addition of 0.5 μM MitoQ-NPs or equivalent volume of blank-NPs, placental explants were incubated for 24 h in Gibco Neurobasal media with 1× B-27 Supplement, 1× antibiotic-antimycotic (Thermo Fisher Scientific) and 2 mM L-glutamine (Sigma-Aldrich).

Conditioned medium samples were randomly selected for analysis (Table 3). Amino acids were measured as previously described [28,29]. Total RNA was isolated from conditioned medium using the miRNeasy Mini Kit (Qiagen, Germany) and levels of individual microRNAs were analysed with the nCounter Human v2 miRNA Expression Assay (NanoString Technologies, U.S.A.). Differential abundance of microRNAs was analysed as previously described [28]. Briefly, unwanted variation was removed with RUVSeq [88] and differentially abundant microRNAs were then predicted using edgeR [89]. Predicted targets of differentially abundant microRNAs were downloaded from TargetScanHuman 7.2 [90] with a context++ score threshold of −0.2. Enrichment analysis of predicted microRNA targets was performed using the GO-slim feature in PANTHER 11.0 [91,92] as well as the UP_TISSUE and KEGG pathway features in DAVID 6.8 [93,94]. Generated data files have been deposited in NCBI’s Gene Expression Omnibus [95] (accession number GSE110786).

### Cortical cultures

Mixed, astrocyte-only and neuron-only cortical cultures were prepared from dissociated rat E18 cortical tissue [29]. The effect of glutamate receptor inhibition was investigated by adding 10 μM MK801 to the cultures 4 h before incubation with conditioned media. Immunocytochemical assessment was performed as described previously [28] using antibodies against MAP2 (Synaptic Systems 188004; 1:2000), GFAP (CST 3670; 1:1000), tyrosine hydroxylase (abcam ab112; 1:500), GluN1 (Millipore Ab9864; 1:200), GluN3α (Millipore 07-356; 1:200), GABA Aα1 (abcam ab33299; 1:250) and GABA B1 (abcam ab55051; 1:250).

### Statistics

Two-way ANOVA was performed in Prism 6.0 (GraphPad, U.S.A.) or SPSS 21.0 (IBM Corp., U.S.A.) with *post-hoc* analysis using Bonferroni correction or Tukey’s test for multiple comparisons.

## Supplementary Material

Supplementary Table S1

## Abbreviations

GABA: γ-aminobutyric acid
NMDA: *N*-methyl-d-aspartate
NP: nanoparticles
PE: preeclampsia
TH+: tyrosine hydroxylase-positive
